# Covered Stent Placement for a Splenic Artery Pseudoaneurysm in a Patient With Inflammatory Vascular Disease

**DOI:** 10.7759/cureus.116138

**Published:** 2026-09-12

**Authors:** Anirudh Maddali, Aidan Farrell, Lawrence Keating

**Affiliations:** 1 Neurosurgery, Hackensack Meridian School of Medicine, Nutley, USA; 2 Interventional Radiology, Albany Medical Center Hospital, Albany, USA

**Keywords:** embolization, leukocytoclastic vasculitis, pancreatitis, splenic artery pseudoaneurysm, stent placement, systemic lupus erythematosus

## Abstract

Splenic artery pseudoaneurysms (SAPs) are potentially life-threatening vascular lesions with a high risk of rupture that may be associated with inflammatory vascular pathologies. While transcatheter embolization is the most common endovascular treatment for SAPs, covered stent placement preserves arterial patency and splenic perfusion but is limited by anatomic constraints, such as inadequate landing zones and excessive vessel tortuosity. This case report discusses the clinical and technical considerations in successfully stenting a SAP in a patient with a history of systemic lupus erythematosus with type III cryoglobulinemia, biopsy-proven leukocytoclastic vasculitis, and necrotizing pancreatitis.

## Introduction

Splenic artery aneurysms (SAAs) or pseudoaneurysms (SAPs) are the most common visceral artery aneurysms, accounting for 60-70% of cases [1]. While true aneurysms involve all three layers of the arterial wall (intima, media, and adventitia) and are associated with connective tissue disorders, portal hypertension, and pregnancy, pseudoaneurysms are contained ruptures of the vessel wall that can occur from iatrogenic trauma or inflammatory pathologies like chronic or necrotizing pancreatitis [2]. Typically, a splenic pseudoaneurysm presents with pain and can be seen on imaging as the splenic artery and intrasplenic branches surrounded by a hematoma [2]. Due to the lack of a true wall, pseudoaneurysms are more prone to rupture. The Society for Vascular Surgery Clinical Practice Guidelines strongly recommends the treatment of all SAPs of any size in patients with acceptable risk due to the possibility of rupture, as well as emergent intervention for any ruptured lesions [3].

Historically, treatment of SAAs and SAPs involved open surgical repair [3]. However, management has shifted towards minimally invasive options such as covered stent-graft placement and embolization due to shorter hospital stays and lower rates of cardiovascular complications [4]. Embolization is the most common approach for treating SAAs and SAPs, typically using coils or liquid embolic agents [5]. While embolization is considered to be an effective form of management, there are certain risks associated with this procedure, including vessel sacrifice potentially causing splenic infarction [5].

Covered stent-graft placement offers an alternative endovascular approach with the potential benefit of preserving arterial flow while excluding SAAs or SAPs [6]. Specific patient populations in which splenic vascular preservation is required include those with portal hypertension, liver transplant candidates, and patients with underlying hematologic conditions in which splenic function is critical [7]. However, stent-graft placement requires specific anatomical criteria. These criteria are defined as adequate proximal and distal landing zones of 10-15 mm and vessel diameters of 4-11 mm, which may be limited by excessive vessel tortuosity, a common feature of the splenic artery [8]. Specifically, distal SAPs or SAPs with excessive vessel tortuosity are generally not suitable for stent-graft placement and may require embolization or open surgery [8].

In this report, we present a case of a patient with a history of systemic lupus erythematosus with type III cryoglobulinemia, biopsy-proven leukocytoclastic vasculitis, and necrotizing pancreatitis with a 3 mm SAP with imaging findings concerning for active hemorrhage, successfully treated with a VIABAHN® 7 mm × 50 mm self-expanding covered stent (W. L. Gore & Associates, Flagstaff, AZ, USA), highlighting consideration of stent placement for patients who may not fit the traditional anatomic criteria for this procedure. This case also supports the potential role of preservative endovascular strategies in inflammatory vascular pathologies.

## Case presentation

A 50-year-old female patient with past medical history of systemic lupus erythematosus with type III cryoglobulinemia, biopsy-proven leukocytoclastic vasculitis, and necrotizing pancreatitis was transferred from an outside hospital for plasmapheresis evaluation due to worsening lupus. Her hospital course was initially notable for hypotension with systolic blood pressure as low as 88 mmHg, tachycardia into the low 100s, and a hemoglobin drop from 9.6 to 7.8 g/dL (reference range: 11.0-14.7 g/dL) in 24 hours. These findings prompted a CT angiography of the chest, abdomen, and pelvis. This study demonstrated necrotizing pancreatitis and multiple intraabdominal collections, most notably a 4.0 × 3.6 cm complex cyst within the pancreatic tail (Figure 1). A 0.3 cm SAP was also discovered, with linear arterial enhancement into the adjacent collection, raising concern for active arterial hemorrhage (Figure 2).

**Figure 1:**
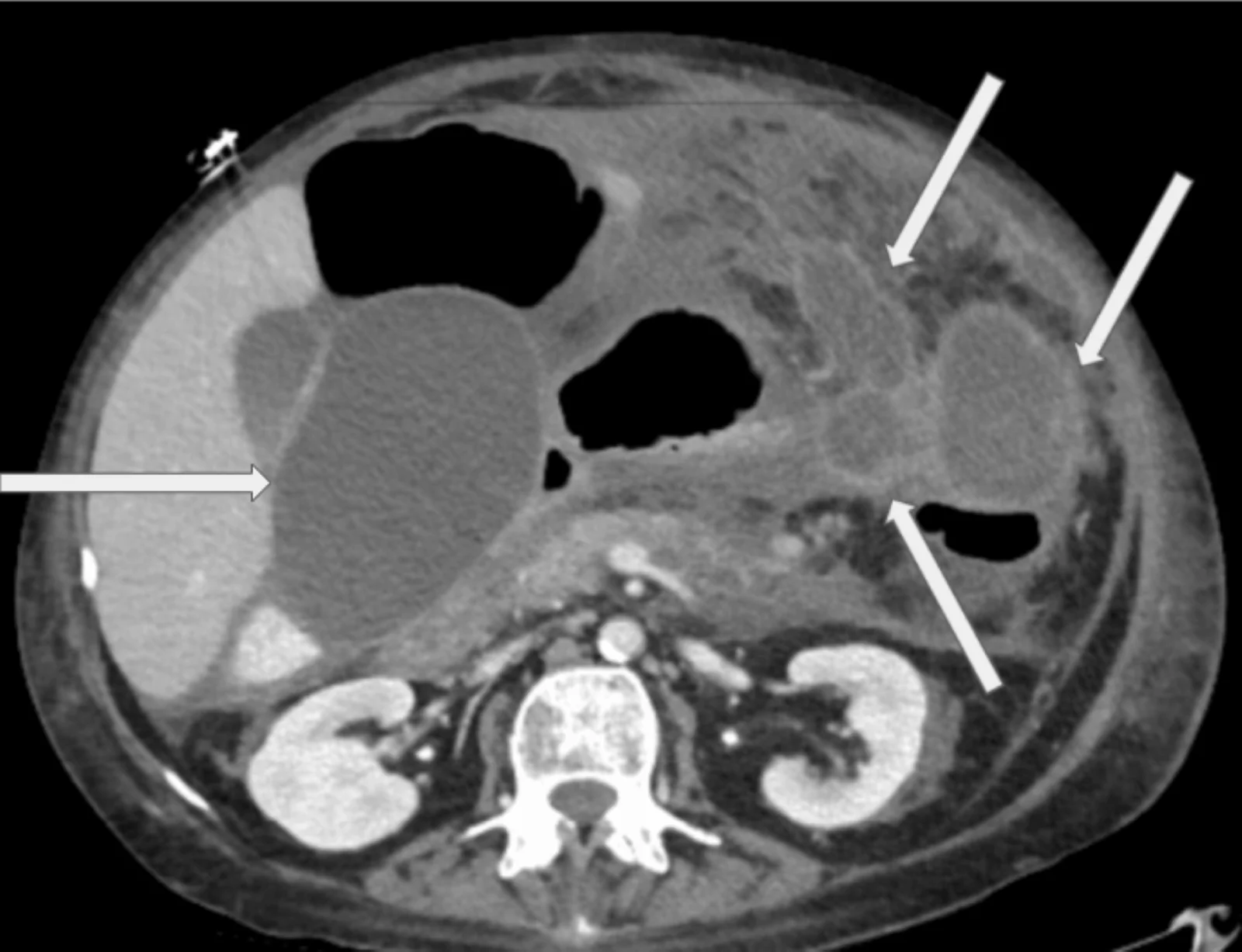
Axial CTA of the abdomen and pelvis demonstrating several well-defined intraabdominal fluid collections (white arrows) and significant intraabdominal inflammatory change. CTA: computed tomography angiography.

**Figure 2:**
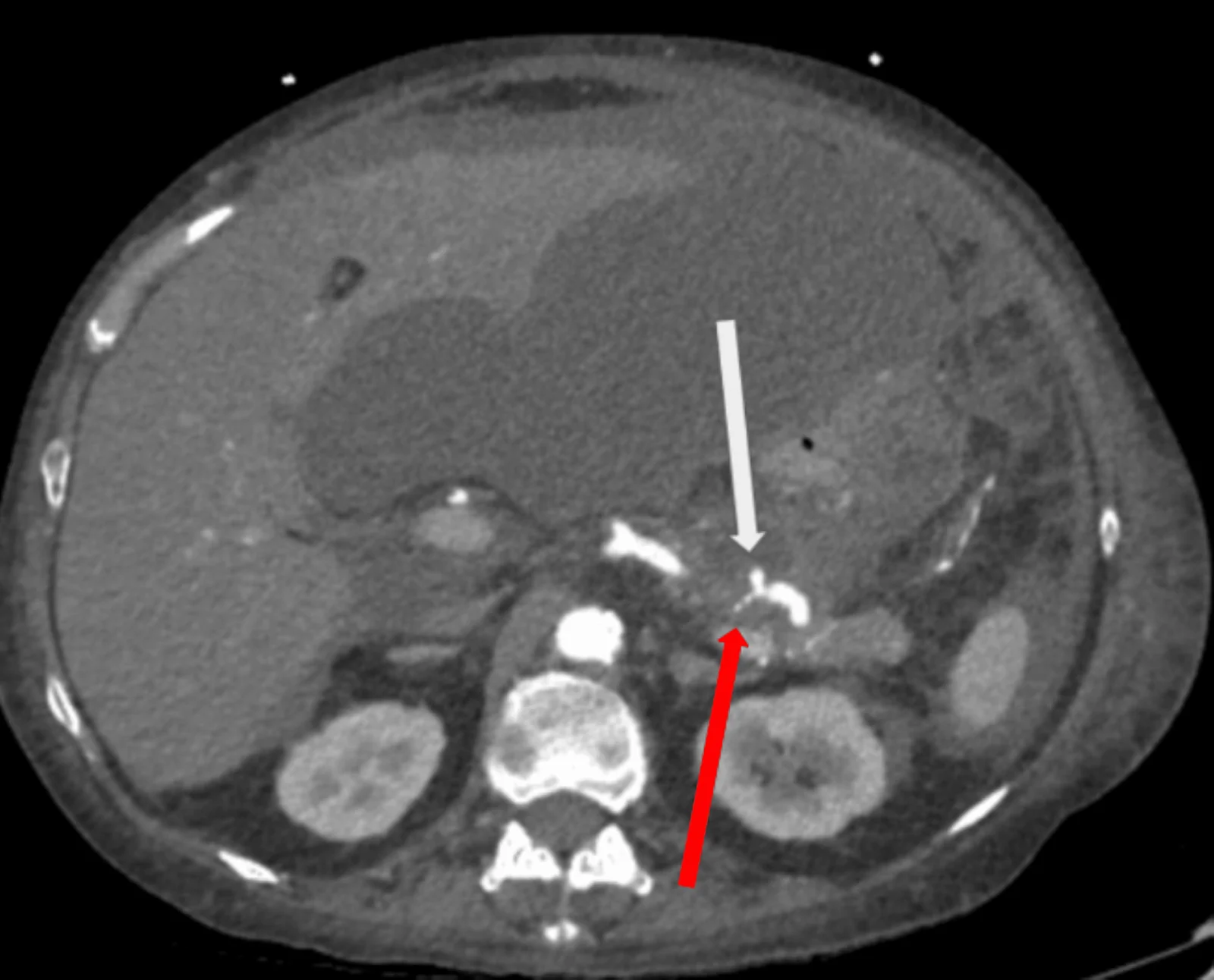
Axial CTA of the abdomen and pelvis demonstrating a 3 mm pseudoaneurysm in the proximal splenic artery (white arrow). A linear hyperdensity adjacent to the pseudoaneurysm raises concerns for active arterial extravasation (red arrow). CTA: computed tomography angiography.

Interventional radiology was consulted for splenic artery angiography and possible splenic artery embolization. The right femoral artery was accessed with a micropuncture set using a 21-gauge needle, and a 7-Fr sheath was placed. The splenic artery was catheterized using a 5-Fr GLIDECATH® Cobra angiographic catheter (Terumo Medical Corporation, Somerset, NJ, USA), and splenic artery angiography was performed, identifying the small pseudoaneurysm in the proximal splenic artery (Figure 3).

**Figure 3:**
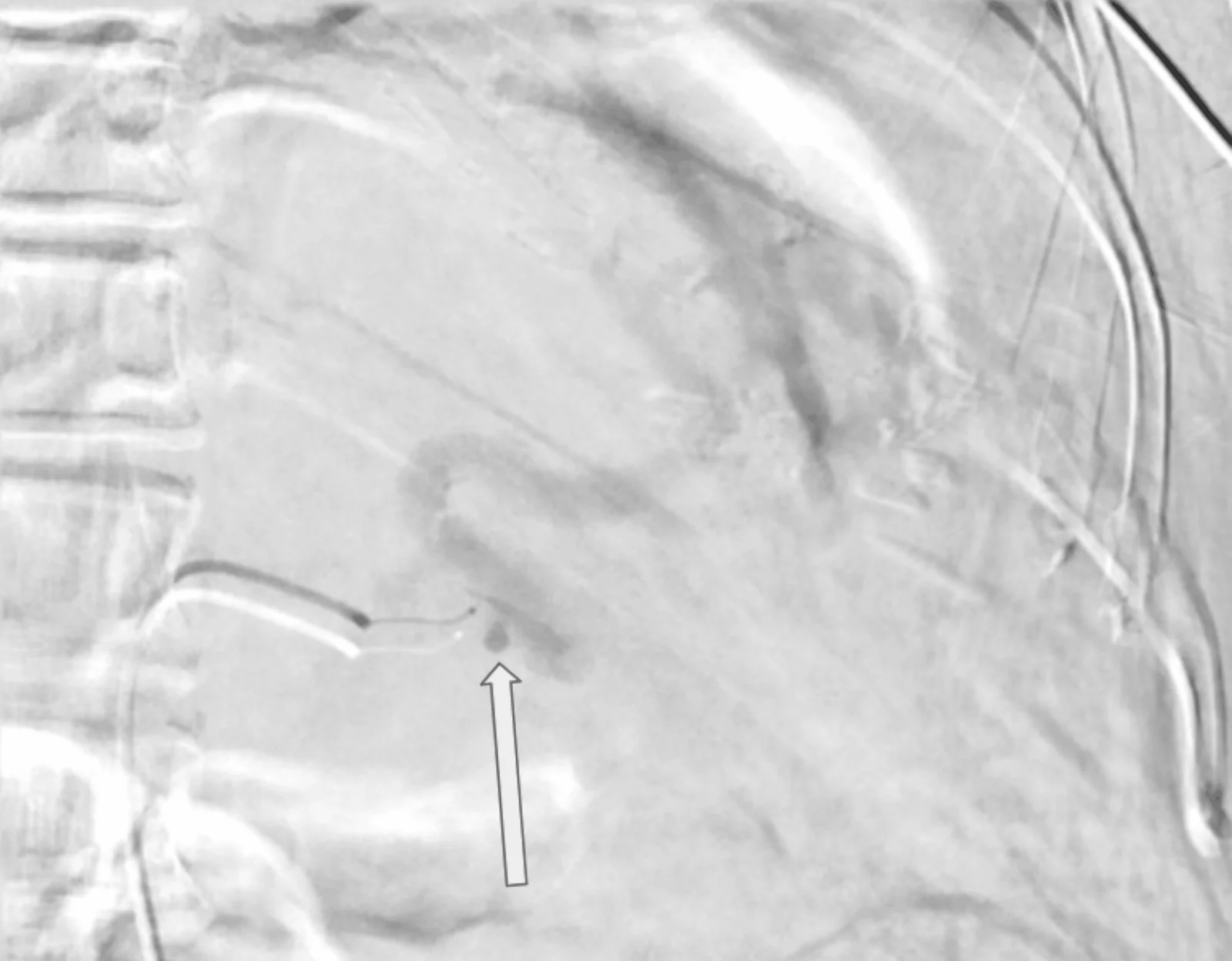
Splenic artery angiogram demonstrating a small proximal pseudoaneurysm (white arrow).

Due to the patient’s chronic pancreatitis and persistent intra-abdominal collections, the SAP was covered with a stent rather than performing an embolization. A VIABAHN® 7 mm × 50 mm self-expanding stent was deployed, successfully preventing blood flow into the pseudoaneurysm (Figure 4).

**Figure 4:**
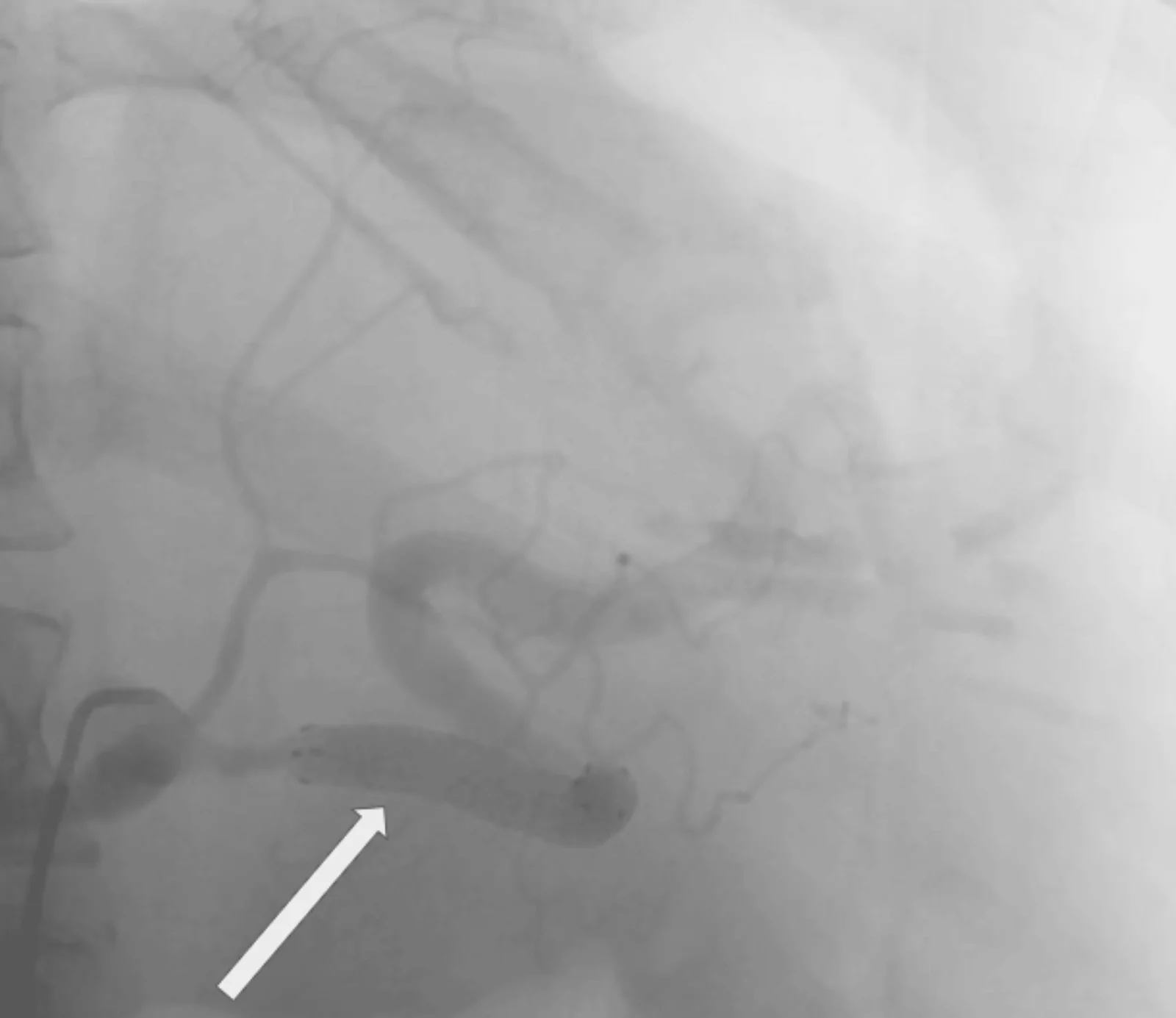
Splenic angiogram demonstrating placement of a VIABAHN® 7 mm × 50 mm self-expanding stent with coverage of the pseudoaneurysm (white arrow).

At the conclusion of the procedure, hemostasis was obtained with manual pressure. The procedure was well tolerated, and the patient left the recovery area in stable condition.

## Discussion

A common factor in SAPs is inflammatory pathologies due to their ability to cause arterial degeneration. Specifically, in pancreatitis, it is thought that necrotizing arteritis leads to arterial wall erosion and fragmentation due to the release of proteolytic pancreatic enzymes [8]. In general, there is up to a 10% chance of spontaneous rupture of a splenic pseudoaneurysm, with this risk increasing to up to 40% when the pseudoaneurysm diameter reaches 20 mm [8]. Because of this risk, appropriate management is crucial. This case demonstrates the effectiveness of stenting in treating a SAP in a patient with a complex systemic inflammatory condition. This case also exhibits the feasibility of preservative endovascular strategies in emergent cases over the vessel-sacrificing alternatives of splenic artery embolization.

The most common percutaneous endovascular treatment for SAAs and SAPs is coil embolization [9]. Embolization occurs simply through one of two mechanisms: delivery of coils into the aneurysm itself or to regions of the splenic artery proximal and distal to the aneurysm to block vascular supply to the lesion. However, there are important disadvantages to consider when evaluating the feasibility of coil embolization over other percutaneous endovascular interventions for SAAs and SAPs. Total embolization of the splenic artery in cases of large or multiple aneurysms can result in splenic infarction [9]. According to previous literature, most splenic infarctions occurred after distal SAA treatment [10]. Another disadvantage can be possible recurrence or relapse of the aneurysm due to incomplete embolization [11]. A more specific disadvantage of embolization in this patient with systemic lupus erythematosus and chronic necrotizing pancreatitis is aneurysm recurrence due to the splenic artery being in close involvement with the patient’s substantial intraabdominal inflammatory processes and the possibility of future vasculitis of the splenic artery.

Covered stent grafts function to exclude SAAs and SAPs from the vascular supply while maintaining blood flow to the rest of the splenic artery. With maintained blood flow, vascular access to the distal splenic artery is maintained if a future aneurysm requires intervention. Other than preserving access for future intervention, stent placement can also be considered for pseudoaneurysms in the proximal to mid splenic artery in an effort to preserve normal splenic perfusion and reduce the risk of splenic infarction [12]. Collateral vessels such as the short gastric arteries can often provide adequate splenic perfusion, but splenic infarction remains a possible complication with splenic artery embolization.

Stent placement was ideal for this patient’s presentation; however, it is important to consider the difficulty of stent graft placements due to the tortuous nature of splenic arteries [13]. In this case, the pseudoaneurysm was located in a non-tortuous segment of the splenic artery, allowing for adequate proximal and distal landing zones for the stent. In a patient with favorable anatomy for stent placement, there are numerous stent sizes available. The diameter of the splenic artery was approximately 6.3 mm in this patient, so a slightly oversized 7-mm-diameter stent was chosen to ensure an adequate seal and minimize the risk of stent migration. The length of the stent was 50 mm, ensuring an adequate amount of stent within the landing zones proximal and distal to the lesion. When these landing zones are limited by excessive vessel tortuosity, coil embolization or open surgery is preferred. Wang et al. came to similar conclusions after placing stents in 11 patients with SAAs. They had no recorded complications with their stent placements and concluded it is the preferred treatment in patients with favorable anatomy, as it maintained normal physiologic splenic perfusion and was a practical, cost-effective way to treat these patients [12].

## Conclusions

SAPs carry a significant risk of rupture and should be treated without delay. Embolization is the most common approach for minimally invasive management of pseudoaneurysms, although stent placement is another management option. Stent placement is a valuable option, as it preserves distal arterial flow and reduces the risk of splenic infarction. In other scenarios, such as in patients with inflammatory conditions like chronic pancreatitis, splenic artery stenting is valuable for allowing continued access to the splenic artery if future splenic artery damage or a repeat pseudoaneurysm were to occur. This case demonstrates the importance of considering future complications of procedures in patients with specific comorbidities and also demonstrates the strengths and limitations to splenic artery embolization and stent placement.
